# The stage analysis and countermeasures of coal spontaneous combustion based on “five stages” division

**DOI:** 10.1371/journal.pone.0202724

**Published:** 2018-08-23

**Authors:** Hongqing Zhu, Kai Sheng, Yilong Zhang, Shuhao Fang, Yunlong Wu

**Affiliations:** 1 School of Resources and Safety Engineering, China University of Mining & Technology (Beijing), Beijing, China; 2 State Key Laboratory of Coal Resources and Safe Mining, China University of Mining & Technology (Beijing), Beijing, China; Heidelberg University, UNITED STATES

## Abstract

The “three stages” division of coal spontaneous combustion is fuzzy and lacks adequate risk and warning levels corresponding to its divisions; additionally, the targeted prevention measures for each stage have not been described. To address the shortcomings of the “three stages” division, the “five stages” division was proposed to more clearly analyze the stage changes of the spontaneous combustion of coal. The “five stages” method divides the process of the spontaneous combustion of coal into five stages, including: the latent stage, heat accumulating stage, evaporation stage, active stage, and hypoxic stage. The critical point of each stage was determined using adiabatic oxidation experiments and programmed heat experiments. As the critical point of the latent stage, the temperature of zero activation energy is approximately 55–70°C. In the heat accumulating stage, the critical point is the temperature (approximately 90°C) where the external moisture of coal evaporates violently while the internal moisture of coal has not yet fully evaporated. During the evaporation stage, the temperature (approximately 105°C) where the internal moisture has evaporated completely represents the end of this stage and the start of the active stage (105–170°C). When the oxygen concentration drops to 5%, the spontaneous combustion of coal enters the hypoxic stage. Thus, an oxygen concentration of 5% represents the critical point of the start of the hypoxic stage (above 170°C). After the analysis of each stage, risk and warning levels were determined. Considering the major prevention measures of the spontaneous combustion of coal, a staged warning and disposal table was created.

## Introduction

The mechanism of the spontaneous combustion of coal has been studied since the seventeenth century and, to date, a number of coal spontaneous combustion theories have been proposed. These include: the pyrite-related cause theory, the bacteria-related cause theory, the phenolic-groups-related cause theory and the coal-oxygen-compound theory [1]. Among them, the coal-oxygen-compound theory has been widely accepted by scholars because the adsorption of oxygen to coal and the production of an exotherm, which is core of this theory, has been proven by experiments. Some scholars [2–6] have supported this theory and described and discussed the mechanisms of the spontaneous combustion of coal as follows: (1) the percolation of air through coal results in a measurable rise in temperature, which is caused by a series of adsorptive, absorptive, and chemical processes; (2) the heat generated gives rise to an increase in the temperature of the coal, which accelerates the rate of coal oxidation; (3) these actions cause the coal to self-heat, and if conditions are favorable, spontaneous combustion will occur.

In previous studies of coal-oxygen compound reactions, various parameters of coal spontaneous combustion processes were studied using adiabatic oxidation tests, programmed heat experiments and gas chromatography [3–11]. Based on the coal-oxygen-compound theory, many scholars believe that the phenomenon of coal spontaneous combustion may generally be divided into three stages: the incubation stage, the self-heating stage and the combustion stage. Each stage is separated by variations in temperature and concentrations of the main index gases (CO_2_, CH_4_, H_2_O, CO). Cole [12] discussed a detailed reaction mechanism while researching the influence of pyrite on the spontaneous combustion of coal and noted that mineral transformations were closely related to the generation of free radicals in coal. Based on the “three stages” division, Qu [13] obtained the stage characteristics and critical temperature points of low- and high temperature-stages in the process of the spontaneous combustion of coal using a simultaneous thermal analysis experiment, Fourier transform infrared spectrum analysis and a programmed heat experiment. Xu [14] investigated the heat release characteristics, changes in functional groups and oxygen consumption in the process of coal spontaneous combustion through adiabatic oxidation and Fourier transform infrared spectrometry analysis, and they found that the heat release of coal has obvious segmentation characteristics.

Most previous studies have been based on the “three stages” division and mainly concentrated on the variation laws of characterization parameters, such as oxygen consumption, activation energy, and functional groups, but they have not proposed corresponding disposal measures and warnings for all stages during the process of the spontaneous combustion of coal. The “three stages” division of coal spontaneous combustion is based on the coal-oxygen compound theory, which is fuzzy and contains no explicit division points. The determination of the spontaneous combustion stage often requires testing the concentrations of index gases or assessing temperature changes. Meanwhile, coal with different coal qualities (i.e., different metamorphic grades) have different critical temperatures of spontaneous combustion and a large range of critical temperature values. More practical and universal stage divisions should involve variations in coal characteristics. Thus, here, the authors propose a “five stages” division and determine the limits for each proposed stage. As a practical result, stage warnings and disposal mechanisms are established, which may help the fire prevention departments of coal mines take effective and corresponding preventive measures before a particular stage of coal combustion.

## Methods

### Adiabatic oxidation experiment

**Fig 1:**
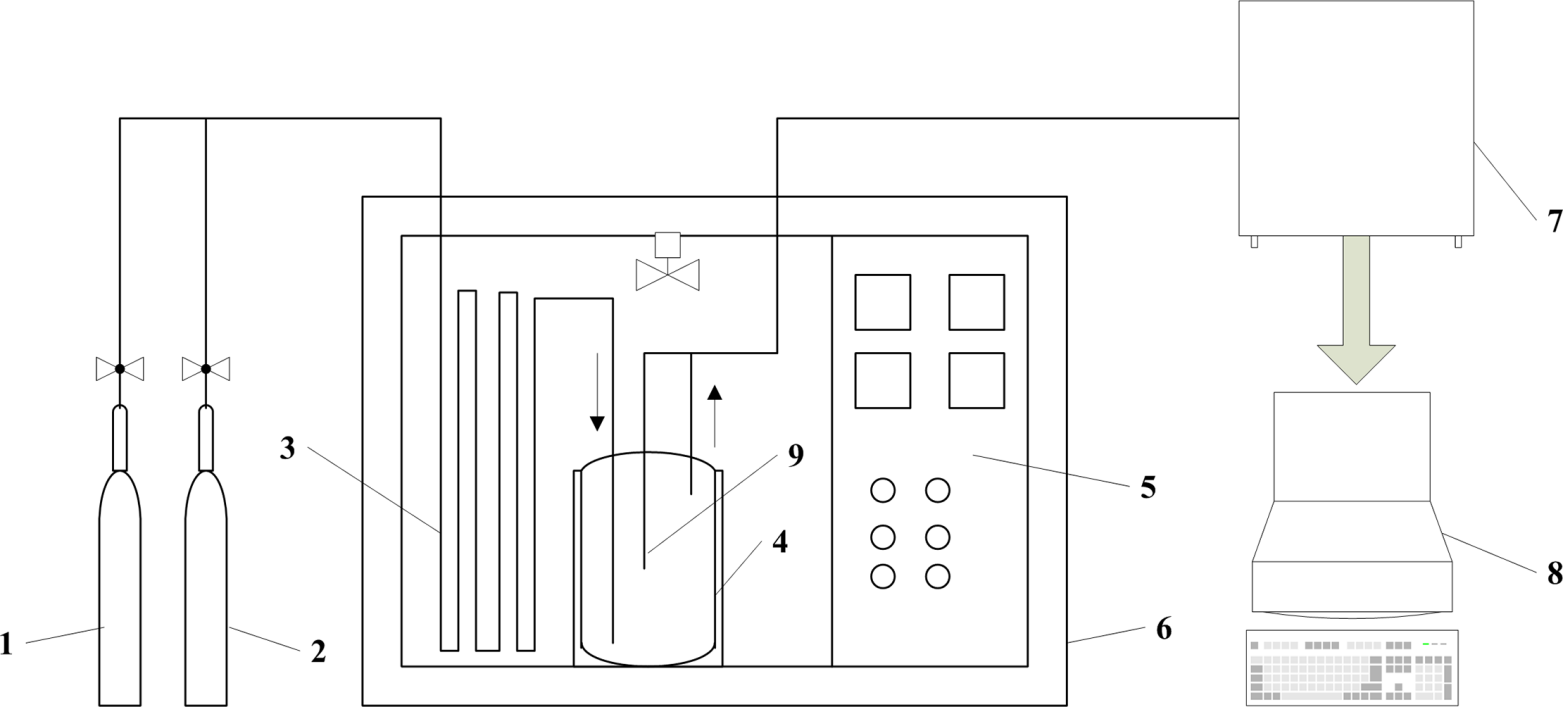
The small coal adiabatic oxidation testing system. 1-Nitrogen cylinder; 2-Oxygen cylinder; 3-Preheating pipeline; 4-Adiabatic tank; 5-Control panel; 6-Adiabatic oven; 7-Gas chromatograph; 8-Computer.

We used an adiabatic oxidation experiment to obtain the temperature of zero activation energy. This experiment uses a small coal adiabatic oxidation test system, which was created at the Safety Engineering Faculty, China University of Mining & Technology (Beijing) and is shown in Fig 1. The entire experiment system consists of a gas source system, gas path, preheat gas system, coal sample tank, adiabatic oven, temperature monitoring system, and data acquisition system.

#### Coal samples

To assess the variable characteristics of the coal, we collected three types of coal samples from the Renlou Coal Mining Co., Ltd (NO.1-3). The proximate analysis of the coal samples were tested, and the results are shown in Table 1. The results indicate that sample NO.1 is a long flame coal, sample NO.2 is a gas coal, and sample NO.3 is a charred coal.

**Table 1:** The industry index values of three coal samples.

Samples	Moisture (%)	Volatile matter (%)	Ash (%)	Fixed carbon (%)
NO.1	8.47	40.62	9.87	41.04
NO.2	19.49	36.35	6.26	37.9
NO.3	1.895	23.515	18.27	56.32

#### Experimental procedures

Prior to the experiment, 200 g of a pulverized coal sample were brought into the tank and dried for 16 hours in a nitrogen-rich atmosphere (with a nitrogen flow rate of 120 mL/min), and the adiabatic oven was set at 105°C. After drying, once the temperature of the coal sample had dropped down to room temperature, the coal sample was transferred into the adiabatic oxidation tank. Then, the temperature program was switched to automatic tracking mode, the nitrogen valve was closed, and the system started pumping in oxygen at a flow rate of 60 mL/min. The computer recorded and saved the changes in coal temperature data over time.

### Isothermal heating experiment

Original coal samples were collected from the Renlou Coal Mining Co., Ltd.; their original moisture content was 2.15%. These samples were then used to make coal samples with water contents of 5.6, 7.2, 9.35, 11.8, 13.1 and 15.25%, and 200 g of each sample were used for the experiment. The experimental device used here is the small coal adiabatic oxidation test system. Including the original coal sample, a total of seven coal samples with different moisture contents were placed into the coal sample tanks; then, the tanks were placed into an adiabatic oven and underwent isothermal heating at 150°C (The oven temperature can be held constant from 40 to 200°C, within an accuracy of ±0.5°C. In each experiment, the oven is first turned on and preset at a certain temperature. After about 30 min, the isothermal condition is reached in the oven.). To avoid the influence of the heat released from the coal oxidation of the coal sample itself, a 120 mL/min flow of nitrogen was inlet into the tanks. A computer recorded the temperature and heating time of each coal sample.

### Programmed heat experiment

#### Coal samples

To assess the different coal qualities, six coal samples (A/B/C/D/E/F) collected from six different places in the Renlou Coal Mining Co., Ltd. were used in the programmed heat experiment. The proximate analysis of the coal samples were tested, and the results are shown in Table 2. These data suggest that the coal qualities of the six coal samples differ.

**Table 2:** The proximate analysis test results.

Coal samples	Moisture (%)	Volatile matter (%)	Ash (%)	Fixed carbon (%)	Sulphur (%)
Sample A	1.53	26.21	19.22	53.74	1.174
Sample B	1.67	27.01	22.32	49.82	0.364
Sample C	3.62	25.02	29.76	43.54	0.359
Sample D	1.33	28.60	14.88	55.75	0.258
Sample E	1.91	29.10	18.92	50.10	0.610
Sample F	1.89	30.15	9.99	58.05	0.496

#### Experimental procedures

The coal samples were crushed under the protection of nitrogen, and a total of 200 g (in 40-g aliquots) were sieved to particle sizes of 1.23–1.6, 1.6–2.5, 2.5–3.5, 3.5–5, and 5–7 mm. After obtaining uniform mixtures, the coal samples were placed into a tank in a nitrogen-rich atmosphere (with a nitrogen flow rate of 120 mL/min). The tanks containing coal samples were placed in an adiabatic oven to dry for 15 h at 105°C. After the completion of the drying procedure, the program turned off the nitrogen and opened an air valve (at the same rate as above) for the programmed heat experiment. The compositions of gaseous products were analyzed at each 10°C step by gas chromatography. The temperature rising program was set as follows: the temperature first remained at 25°C for 30 min, then increased to 35°C in 1 min, and finally rose to 185°C at a rate of 0.5°C/min. When the temperature reached 200°C, the experiment ended.

## "Five stages" of coal spontaneous combustion

### The latent stage

Here, the time interval from the exposure of the coal seam to the air to the beginning of the increase in coal temperature is called the latent stage. This stage includes two substages, and the first one is the traditional latent stage. The main reaction between coal and oxygen is physical adsorption, which releases a small amount of heat and adsorbs oxygen to form unstable oxides or oxygen-containing free radicals. The second substage is the preparation stage, which leads up to a critical point of the heat accumulating stage. In the preparation stage, the chemical adsorption increases and heat production is greater than heat dissipation; at the same time, the coal temperature gradually increases at a lower rate. In this stage, CO and CH_4_ usually appear and slowly increase, CO_2_ slowly increases, and oxygen essentially remains stable [13].

According to the coal-oxygen-compound theory, coal is a porous medium, and the strong adsorption of air may occur within it. The original heat production is mainly due to the physical and chemical adsorption of oxygen on coal at the beginning of the spontaneous combustion of coal. The basic dynamic equation in the process of the spontaneous combustion of coal can be described as follows [15,16]:

$$c\rho \frac{\partial T}{\partial t}=Q\rho A\exp\left(-\frac{E}{RT}\right)+\lambda \nabla^{2}T-c\rho_{oxygen}v\frac{\partial T}{\partial x}-H_{w}\frac{\partial C_{w}}{\partial t}$$ (1)

In the above equation, *c* is the specific heat capacity of the sample material, J kg^-1^ K^-1^; *ρ* is the density of the sample, kg m^-3^; *T* is the sample temperature, K; ***t*** represents time, s; *Q* is the heat of oxidation per unit mass under standard state conditions, kJ kg^-1^; *A* refers to the former factor, s^-1^; *E* is the activation energy, kJ mol^-1^; *R* is the ideal gas constant, 8.314 J K^-1^ mol^-1^; *ν* is the flow rate of oxygen, m s^-1^; *H*_*w*_ is either dry or humid heat, J m^-3^ s^-1^; *λ* is the coefficient of heat conduction, W m^-1^K^-1^; and *C*_*w*_ is the moisture content of coal, %.

There are four terms on the right side of Eq (1); the 2^nd^, 3^rd^, and 4^th^ terms represent heat conduction, heat convection and transfer and water evaporation, respectively. These are all external factors of the spontaneous combustion of coal. The first term is the kinetic expression of the heat produced by the oxidation of coal at low temperatures, which is the inner driving power of coal spontaneous combustion. In the latent stage, coal is at a low temperature similar to the surrounding temperature; thus, the 2-4^th^ terms can be ignored. Eq (1) can be simplified as Eq (2) [16,17]:

$$c\rho \frac{\partial T}{\partial t}=Q\rho A\exp\left(-\frac{E}{RT}\right)$$ (2)

Eq (3) can be obtained by the logarithm transformation and simplification of Eq (2) [16,17]:

$$\ln\left(\frac{\partial T}{\partial t}\right)=-\frac{E}{RT}+\ln\left(\frac{QA}{c}\right)$$ (3)

The activation energy *E* is the core parameter of coal low-temperature oxidation power, on behalf of the low-temperature oxidation capacity, namely, the coal spontaneous combustion tendency. Therefore, the change in activation energy should be regarded as the critical index of the latent stage. Lu [18] tested the range of different coal samples using an adiabatic oxidation experiment and obtained a relationship between activation energy and temperature. In the thermal range of 55–70°C, the activation energy of the coal sample changes from positive to negative [18,19]. This means that the activation energy exists at a zero value in the given range. Different coal samples reach zero activation energy at different temperatures. The zero activation energy theory explains the process by which the coal gains heat from physical and chemical adsorption until it can produce heat itself. Therefore, the zero activation energy [20] can be calculated to determine the critical point between the latent stage and the heat accumulating stage.

**Fig 2:**
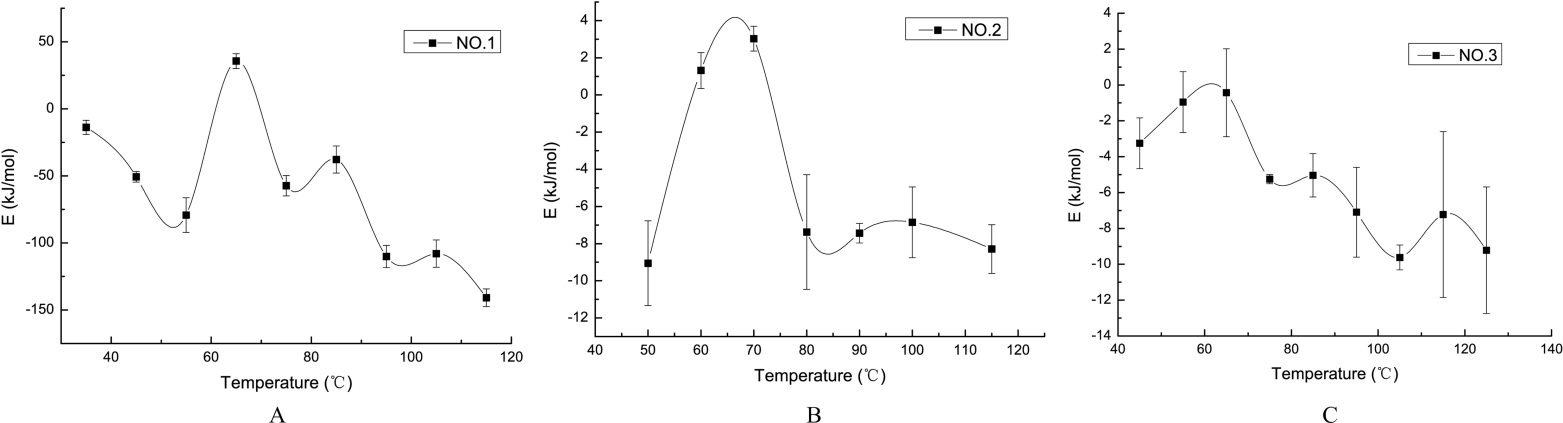
The temperature-dependent activation energy curve.

We also used adiabatic oxidation experiments to obtain the temperature of zero activation energy. Based on our experiment, we calculated the activation energy of the coal samples when the temperature increased at each 10°C step to obtain the temperature-dependent activation energy curve, as shown in Fig 2.

Fig 2 shows that in the range of 55–70°C, the three different coal samples reach their zero activation energy at approximately 60°C, and their activation energy tends to decrease when the temperature exceeds 60°C. Thus, the critical temperatures of the three coal samples in the latent stage are approximately 60°C. Due to the limited number of test samples, 60°C may not be the critical temperature for other coal samples in the latent stage. The results of other researchers also suggest that the zero activation energy often occurs in the range of 55–70°C; the exact value relies on the quality of the coal [19].

### The heat accumulating stage

The period from the active oxidation heat release to the point at which water evaporates violently is the second stage of the spontaneous combustion of coal. Here, this is called the heat accumulating stage. At this stage, the heat release from chemical adsorption still plays a considerable role, but the oxidation heat release rates of coal and oxygen increase rapidly, and the CO and CO_2_ emissions continue to increase. During this stage, the external water of coal evaporates faster with the increasing temperature, while the internal water does not evaporate yet. The steam generated by the evaporation of external water will remove some heat, but it may be partially absorbed by coal pores that will retain the heat and fill the coal pores, thus producing a heat-preserving effect similar to the “greenhouse effect”. Beamish [19] found that a coal sample with a higher moisture content needs more time to cool down from 110°C to 40°C. This demonstrates the existence of a heat preservation effect in coal related to moisture evaporation. During the early stage of the spontaneous combustion of coal, external moisture begins to evaporate (at approximately 50°C) with a catalytic action [21–26], because moisture is involved in the formation of free radicals and plays an important role in promoting the formation of peroxide complexes [27]. Water evaporation in coal can also lead to the decomposition of peroxide complexes that will accelerate combustion. Meanwhile, due to the dynamic action of water vapor, many pores or fissures will be formed within coal, so that the surface area of coal and the contact area between coal and oxygen will increase. At approximately 40–50% of the moisture holding capacity of the coal, above this critical level of moisture content, the heat produced by oxidation is dissipated by moisture evaporation and coal self-heating is significantly delayed [19]. When the moisture content is higher than a critical value, a layer of a water-containing liquid membrane will form at the coal surface. This membrane may hinder the contact of coal and oxygen and prevent their interaction. This situation is, however, rare. Normally, the moisture content in coal is lower than the critical value.

**Fig 3:**
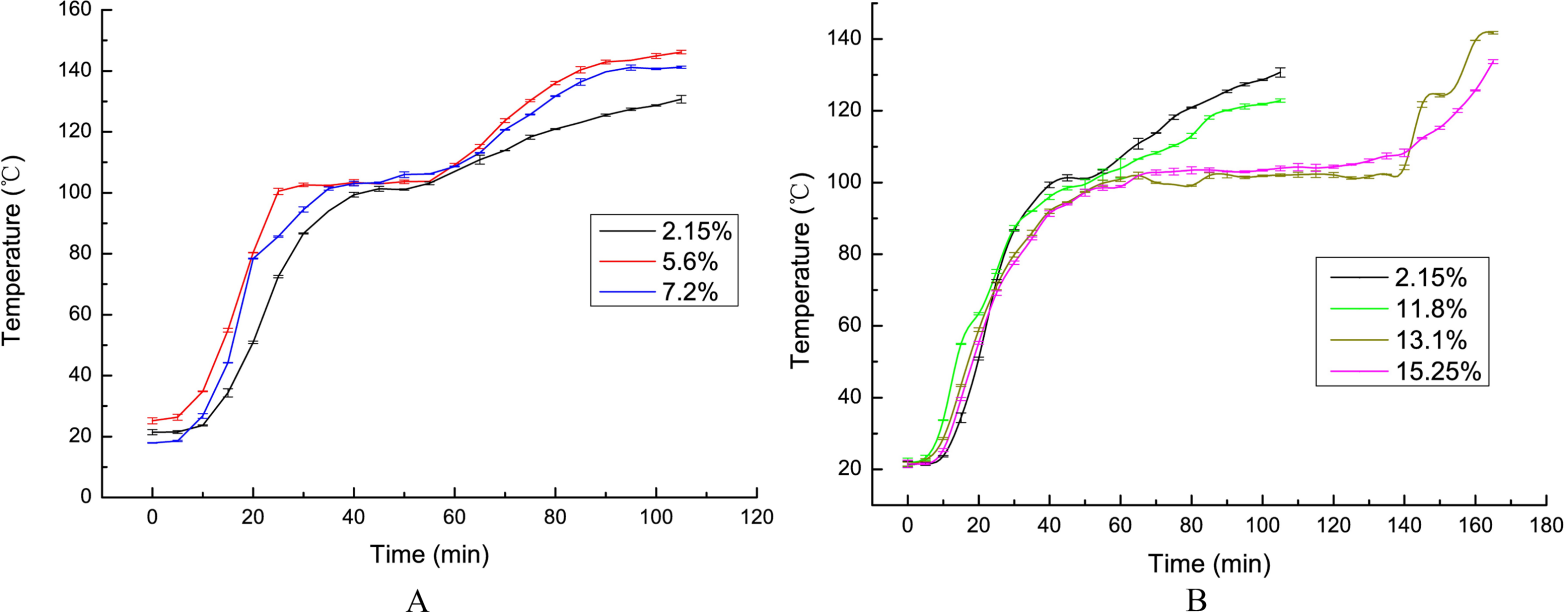
Thermal curves of temperatures of coal samples with different moisture contents. (A) The coal samples with water content of 5.6% and 7.2% were plotted together with original coal sample. (B) The coal samples with water content of 9.35%, 11.8%, 13.1%, and 15.25% were plotted together with the original coal sample.

To find the critical temperature point of the heat accumulating stage, we must determine the starting point at which the moisture content has an important impact on the heating process of coal. Therefore, we designed an isothermal heating experiment for coal samples with different moisture contents. Based on the data obtained, the temperature curves of coal samples with different moisture contents were drawn, as is shown in Fig 3.

There is a huge internal surface area in the coal. After water evaporation from the coal, more pore channels or fissures are formed, so that the coal has a larger internal surface area, which is beneficial for more oxygen to enter the coal for internal oxidation. The predominance of the endothermic reaction in the primary stage of coal self-heating is mainly due to the evaporation of moisture in the coal. After the water in the coal evaporates, the oxidation activation center of the coal surface is increased. The second phase after the evaporation of water, that is, after the dehydration phase, the reaction becomes more intense as more points of evaporation water are distributed. According to the study of oxidation under adiabatic conditions (simulating the conditions of the mine), the evaporation of moisture can make the coal oxidation more intense. Initially, the oxidation was carried out in large pores. After the water evaporates, oxidation takes place in the medium and micropores, which have a large surface area, which greatly increases the specific surface area of the reaction and accelerates oxidation.

As seen in Fig 3, the temperatures of coal samples increase rapidly up to 90°C, although the rates of different samples are slightly different. The heating rate of the coal samples becomes slower when the temperature crosses 90°C, and it even stagnates when it reaches 100°C. This phenomenon demonstrates that the moisture contained in coal begins to have a serious impact on the heating process of coal samples at approximately 90°C. The reason for this is that the external moisture of coal samples begins to evaporate intensely at 90°C. Thus, the temperature at which the evaporation of external moisture occurs represents the critical point between the heat accumulating stage and the following evaporation stage. Fig 3A also indicates that the heating rate of the coal samples with a moisture content of 5.6% and 7.2% are the fastest and are greater than that of the original coal sample; thus, the optimum moisture content of a coal sample falls in the range of 2.15–7.2%.

### The evaporation stage

During this stage, due to the heat accumulation in the previous stage, the temperature is increasing, the content of active molecules [18] within the coal has largely increased, their collisions are more frequent, and the reaction rate between coal and oxygen is higher [28]. At approximately 90–105°C, the external water has almost completely evaporated, and the internal water also starts to evaporate. Eq (1) indicates that the heat generation caused by the process of the spontaneous combustion of coal also includes the humid heat of water. However, water is often absent in a coal sample before the adiabatic oxidation experiment because the coal sample has been dried beforehand; thus, the moisture evaporation stage is often ignored in the analysis and research of the adiabatic oxidation of coal. Because this stage appears in a narrow temperature range and has no obvious characteristics, it is easy to ignore. However, the evaporation stage plays an important role in delaying the further active stages of the spontaneous combustion of coal.

As shown in Fig 3, the thermal range at which the heating rate is almost stationary is exactly 90–105°C. The greater the moisture content is, the longer duration of this phase is (i.e., the heating rate is almost stationary). The coal samples with moisture contents of 13.1% and 15.25% show much longer durations of this stage than the other samples. At 105°C, the internal moisture has almost completely evaporated. The temperature stagnation at this stage is due to the fact that the internal moisture captures some of the heat generated by the oxidation of coal, and the heat requirement of the evaporation of internal moisture is much greater than that of external moisture. Therefore, the effect of the evaporation of internal moisture can delay the active stage, thus providing time for workers to prepare prevention measures.

### The active stage

**Fig 4:**
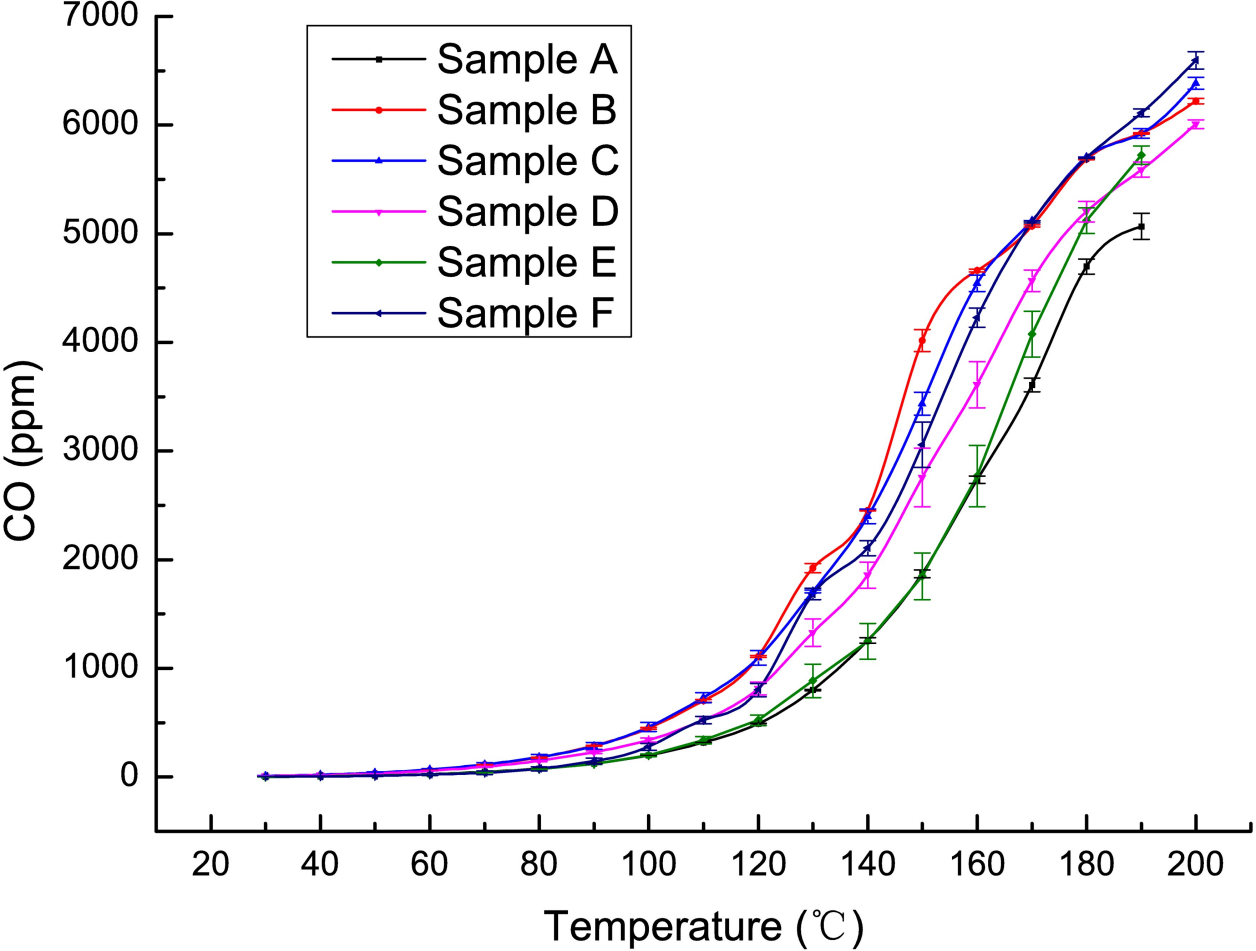
Temperature-dependent variations in CO contents.

**Fig 5:**
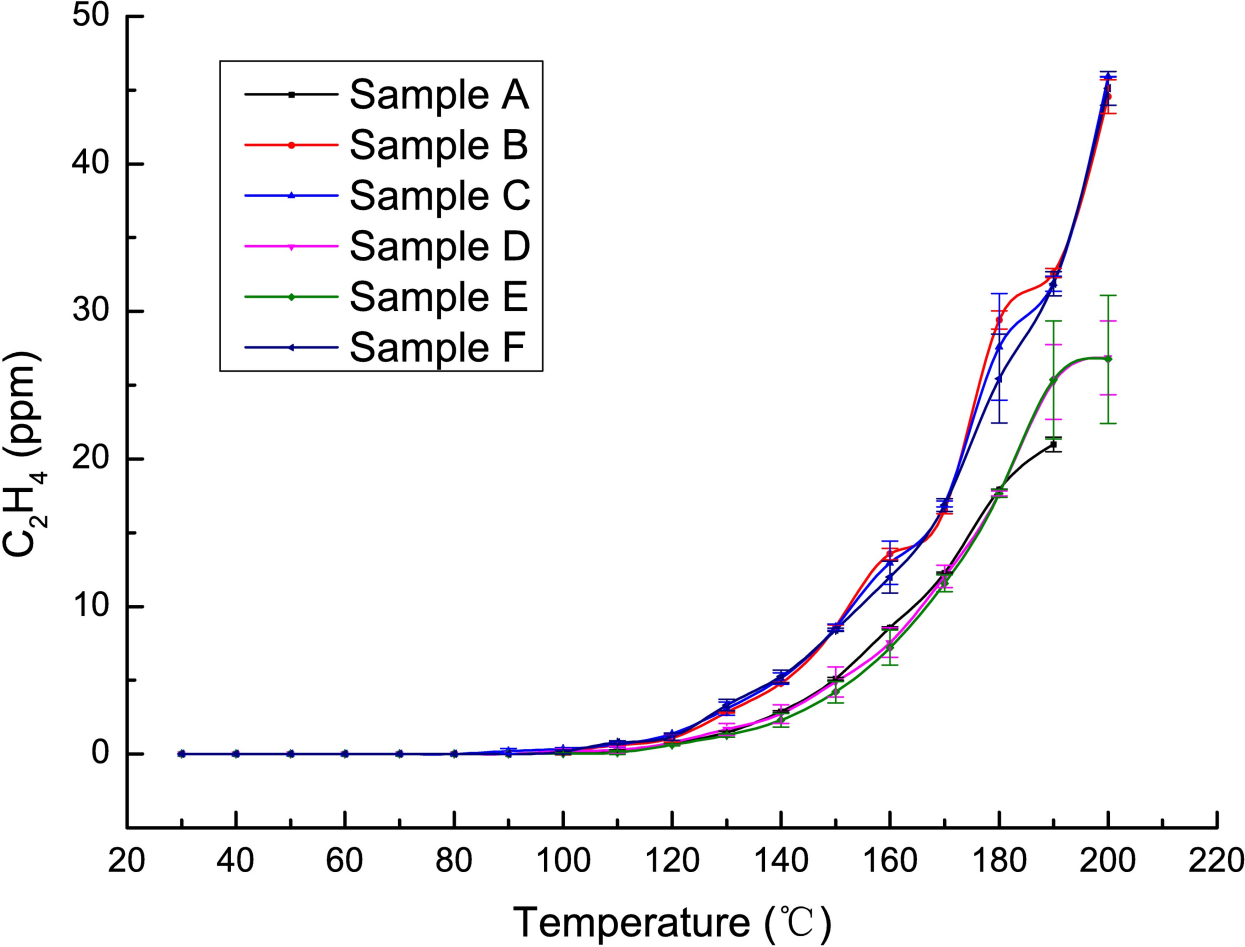
Temperature-dependent variations in C_2_H_4_ contents.

This stage is ascribed to the thermal range of 105–170°C. Due to the complete desorption of both the internal and external moisture in coal during the foregoing stages, the reaction between coal and oxygen begins, and oxygen consumption, heat release and gas production show qualitative changes. Many researchers have proposed that the coal-oxygen recombination reaction occurs in the temperature range of 105–170°C and that lower degrees of coal metamorphism cause faster temperature growth [29–32]. At this stage, the changes in the concentrations of O_2_, CO, C_2_H_4_ and other gases are clearly related to the intensity of the reaction between coal and oxygen. Therefore, a programmed heat experiment for coal samples with different coal qualities was designed to verify the existence of the active stage. The temperature-dependent variations in the contents of CO and C_2_H_4_ are shown in Figs 4 and 5, respectively.

As shown in the above figures, the CO contents in all samples increase rapidly starting at approximately 110°C, while the C_2_H_4_ contents in all samples increase rapidly starting at approximately 115°C. During the spontaneous combustion of coal with different degrees of coalification, there are differences in the consumption rates of O_2_ and the production rates of CO, CO_2_ and C_2_H_4_. Although the critical temperature, *i*.*e*., the temperature at which the rate drastically changes, varies between CO and C_2_H_4_, the critical temperatures of different samples still fall within the range of 100–115°C. This temperature range happens to represent the evaporation temperature at which both the external and internal moisture evaporate completely. Thus, the above analysis demonstrates that when both the external and internal moisture evaporate completely, the reaction between coal and oxygen enters a very active stage because the heat generated by the reaction is no longer used to evaporate moisture.

### The hypoxic stage

**Fig 6:**
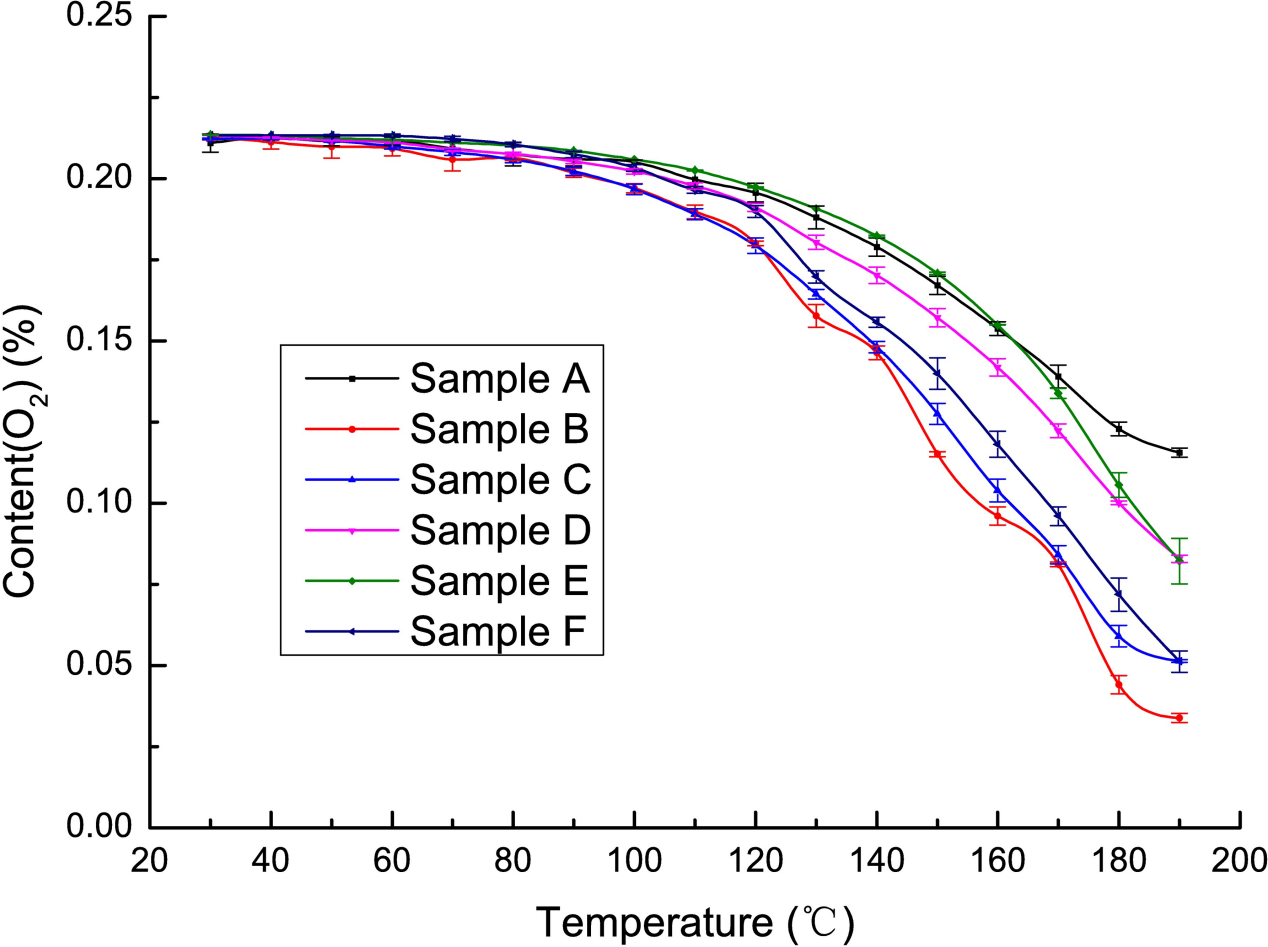
The relationship between oxygen concentration and temperature.

During this stage, two methods of coal combustion may occur. The first occurs when the O_2_ supply is constant, and the second occurs when it increases. The temperatures of the coal samples increase rapidly during the period from the active stage to coal ignition. If the oxygen supply is sufficient, a flame will appear, and the coal sample will start to burn. At this time, a large amount of high-temperature smoke is generated. This smoke contains CO, CO_2_ and hydrocarbons, among other substances [33,34]. However, if the oxygen supply is insufficient, the coal sample will develop smoldering smoke without a flame. In general, as the activated molecules in the coal seam increase with increasing temperature, more oxygen will be required. To maintain the combustion of coal, the supply of oxygen must be increased. However, during the experimental process, the oxygen supply is constant because the oxygen flow is stable. In addition, during the practice of coal mining, the air quantity and air pressure are constant, which means that the air velocity is constant in galleries and that air leakage or flow tends to be constant. Therefore, the oxygen supply for the spontaneous combustion of coal is not expected to increase during the practice of coal mine production. If so, the spontaneous combustion of coal will enter the hypoxic stage. In general, when the oxygen content is less than 5%, the coal in the fire area remains in a smoldering state and flame combustion cannot exist [35]; thus, an oxygen concentration of 5% can be used as a threshold of the spontaneous combustion of coal entering the hypoxic stage. Based on the programmed heating data, the oxygen concentration curves of coal samples A-F are drawn in Fig 6. As seen in this figure, when the oxygen content is approximately 5%, the temperatures of all samples are above 170°C. Thus, the critical temperatures of the hypoxic stage of the six samples are all beyond 170°C. However, the spontaneous combustion characteristics of coal are greatly affected by coal quality, thus affecting the lower thermal limit of the hypoxic stage. The six coal samples assessed in this paper cannot represent all types of coal; thus, the critical temperatures of specific coal samples should be identified using programmed heat experiments, and the effects of coal quality rank on the critical temperature of the hypoxic stage require further study.

## Establishment of staged disposal measures for the spontaneous combustion of coal

### Analysis of various self-ignition prevention applications

The prevention of coal self-ignition mainly include three types of procedures: pressure equalizing, anti-fire material injection and sealing a fire zone.

#### Pressure equalizing

This fire prevention theory suggests equalizing the pressure of a leakage path between an intake and outlet air ventilation stream, with the purpose of either eliminating or reducing the air leakage [36]. The essence of pressure equalizing is to set air pressure regulators or adjust the ventilation system to reduce the pressure between the both ends of the leakage path, thereby reducing access to the coal accumulation area. As a result, one can inhibit or eliminate the coal spontaneous combustion phenomenon. The pressure equalizing technology has the advantages of simple principles and negates the need to ascertain the specific locations of fire sources. It is possible to extinguish a large coal fire using pressure equalizing. The mentioned technology is just an air regulation method that poses no hazard to the mine staff, which requires little expense and has no effect on normal production. In addition, compared with the injection of anti-fire material, pressure equalizing is only related to air, so it is not restricted by water, soil and provenance, and it can continuously prevent fires for a long time in a coal mine. It is applicable to the heat accumulation stage and evaporation stage during the early coal spontaneous combustion process because the scope of the fire is smaller in this period, and fewer harmful gases are produced.

#### Anti-fire material injection

Anti-fire material injection refers to injecting an inert gas, gel material, three-phase foam or other material into the fire area [37–39]. The main purposes of this method are to dilute the oxygen concentration, reduce the temperature and isolate the contact surface between coal and oxygen.

Inert gas injection: The advantages of inert gas injection are that it can cause methane and other flammable gases to lose their explosiveness by reducing the oxygen concentration and that it has no corrosive effect on mining devices or negative effects on the health of mine workers. Its disadvantages include easy gas diffusion, which can be followed by possible air leakage or inert gases spreading randomly, the need for the nitrogen injection machine to be regularly maintained, and its poor cooling extinguishing effect on the fire area.

Gel material injection: The advantages of this method are that it has a good effect on wrapping coal and sealing cracks, the material used is highly temperature-resistant, and the method has better applications to partial or small fires. Its disadvantages are also obvious; it comprises a small-medium flow, has a high cost, and exhibits colloid cracking after a long injection time; additionally, gel interactions with mine brine may produce poisonous gases [40].

Three-phase foam injection: Three-phase foam has the following characteristics [41]: (a) it is formed by injecting nitrogen into a slurry, which leads to a substantial increase in the slurry volume; thus, the volume-expanding slurry forms a large range of coverage in a fire zone. (b) It encapsulates nitrogen so that it can stay in the fire zone longer. (c) It contains fly ash, mud and other solid substances that can provide it with long-term stability, thus allowing it to prevent coal oxidation. (d) Its foaming agent adds retardants that enable it to uniformly disperse through coal and allow it to prevent the formation of coal-oxygen functional groups and the chain reaction of free radicals while also improving the wettability of the coal surface, thus greatly increasing the coal humidity. This method requires less investment and simple equipment; it is not difficult to operate, and it is characterized by continuous high-flow perfusion.

Anti-fire material injection can quickly extinguish a fire and have better extinguishing effects, but it usually requires vast amounts (volumes) of original material for large fire zones, which is very expensive. Thus, this method is suitable for the evaporation stage and active stage because during these stages, fire rapidly develops and the production rates of CO, C_2_H_4_, C_2_H_6_ and other harmful gases increase rapidly; thus, the fire must be quickly controlled.

#### Sealing a fire zone

When a fire has been burning for more than 2 hours, it will be very large and thus difficult to directly extinguish. At this time, the fire zone should immediately be sealed [42].

The essence of sealing a fire zone is reducing or even cutting off the oxygen supply to control or extinguish the coal combustion in a sealed fire zone. This method mainly involves building an airtight wall, which can not only form a sealed zone to cut off the oxygen but may also resist shock waves from gas explosions. Prior to building the airtight seals, one should judge the behavior of the fire based on the concentrations of fire indicator gases (CO, C_2_H_6_, CH_4_, C_2_H_4_, C_2_H_2_) in the fire bundle tube monitoring system. When it is difficult to use direct extinguishment measures to control the fire, more preparations must be done, more human and material resources will be saved, and the fire will be controlled or even extinguished earlier. Therefore, the material preparations for building an airtight wall should be done when the spontaneous coal combustion reaches the active stage.

### Stage warning and disposal mechanisms of the spontaneous combustion of coal

The risk of the spontaneous combustion of coal can be classified based on the following levels: very low risk, low risk, moderate risk, high risk, and extreme risk levels. These correspond to the successive five stages of the spontaneous combustion of coal.

The warning levels are also divided into the following five sublevels: the blue, yellow, primary red, secondary red, and last red warning levels. Based on the analysis of the “five stages” division of the spontaneous combustion of coal and various anti-fire measures in mines, the stage warning and disposal table for the spontaneous combustion of coal is established and shown in Table 3.

**Table 3:** Stage warning and disposal table of the spontaneous combustion of coal.

Stages	Division point	Description	Name	Risk levels	Warning levels	Measures
I		Heat production mainly relies on physical adsorption and chemical adsorption; oxidation reaction of coal transforms from passive to active.	The latent stage	Very low risk	Blue warning	Strengthen monitoring of the spontaneous combustion of coal.
	Temperature corresponding to zero activation energy of coal					
II		Coal-oxygen compound reaction is strengthened, external moisture gradually evaporates and heating rate increases.	The heat accumulating stage	Low risk	Yellow warning	Analyze air leakage and equalize pressure.

	Temperature (about 90°C) at which external moisture evaporates violently but internal moisture has not yet started to evaporate					
III		The duration of this stage depends on the current coal moisture content.	The evaporation stage	Moderate risk	Primary red warning	Equalize pressure, localize fire source, and inject anti-fire material into the fire zone.

	Temperature (approximately 100–115°C) at which internal moisture evaporates completely					
IV		Heat release reaction becomes active; oxygen consumption, heat release and reaction product contents increase dramatically.	The active stage	High risk	Secondary red warning	Drill and inject pertinent anti-fire material, prepare to seal the fire zone.

	The oxygen content drops down to 5%					
V		Sharp increases in temperature and gas product contents; coal spontaneous combustion process enters the hypoxic stage and starts to smolder.	The hypoxic stage	Extreme risk	Last red warning	Seal the fire zone, drill to detect the fire area and inject anti-fire material.


Using the staged warning and disposal table can help workers carry out the targeted fire prevention measures based on the advance level of coal combustion. The prevention measures ascribed to various stages not only consider their practicality and effectiveness based on the stage characteristics but can also reduce their cost and impact on coal production.

## Conclusions

Based on the coal-oxygen-compound theory and due to the shortcomings of the “three stages” division of the phenomenon of the spontaneous combustion of coal, the “five stages” division was proposed; it includes the latent stage, heat accumulating stage, evaporation stage, active stage, and hypoxic stage. Experiments and calculations of the temperature corresponding to the zero activation energy of coal samples determined the critical point between the latent stage and the heat accumulation stage. The temperature of about 90°C, at which the external coal moisture evaporates violently while the internal moisture has not yet evaporated, can be regarded as the critical point between the heat accumulating stage and the evaporation stage. The temperature of about 105°C, at which the internal coal moisture evaporates completely, can be regarded as the ending point of the evaporation stage. After the dehydration of coal, its spontaneous combustion passes to the active stage; during this stage, the temperature of the coal and its related gaseous products increase dramatically. When the oxygen content drops down to 5%, the process enters the final, hypoxic stage. Our six-coal-sample experiments have shown that when the oxygen content is approximately 5%, the temperatures of all samples are above 170°C; thus, the critical temperatures of the hypoxic stage of all six samples are beyond 170°C, and the coal spontaneous combustion characteristics are greatly affected by the quality of the coal, thus affecting the lower thermal limit of the hypoxic stage.

Based on the “five stages” division, the risk levels of the spontaneous combustion of coal are divided into the very low risk, low risk, moderate risk, high risk, and extreme risk levels, and the warning levels of the spontaneous combustion of coal are divided into the blue, yellow, primary red, secondary red, and last red warning levels. Considering the characteristics and applicability of the presented major prevention measures, the staged warning and disposal table for the spontaneous combustion of coal is established corresponding to the characteristics of each hazard and warning level. This work may provide guidance for preventing and controlling the spontaneous combustion of coal at different levels during the development of this process.

## Supporting information

S1 File Data for Figs 2–6. (XLSX)
